# Gene silencing of β-galactosamide α-2,6-sialyltransferase 1 inhibits human influenza virus infection of airway epithelial cells

**DOI:** 10.1186/1471-2180-14-78

**Published:** 2014-03-27

**Authors:** Dong Wu, Wenbo Huang, Yutao Wang, Wenda Guan, Runfeng Li, Zifeng Yang, Nanshan Zhong

**Affiliations:** 1 State Key Laboratory of Respiratory Disease, First Affiliated Hospital of Guangzhou Medical University, 1st KangDa Road, Guangzhou, China; 2 Department of Respiratory, Affiliated Hospital of Guangdong Medical College, South 57, Renmin Avenue, Zhanjiang, China

**Keywords:** Influenza virus, Receptors, Sialyltransferase, RNAi

## Abstract

**Background:**

Human influenza virus hemagglutinin prefers to use sialic acid (SA) receptors via α-2,6 linkages. The β-galactoside α-2,6-sialyltransferase I (ST6Gal I) protein is encoded by the ST6GAL1 gene and is responsible for the addition of α-2,6 linked SA to the Galβ1-4GlcNAc disaccharide of glycans and glycoproteins found on the cellular surface. Therefore, ST6GAL1 could be a potential target for anti-influenza therapeutics. We used specific small interfering RNAs (siRNAs) to block expression of ST6GAL1 and limit distribution of SA receptors on the surface of airway epithelial cells.

**Results:**

The siRNA duplexes we used inhibited ST6GAL1 mRNA expression and subsequent expression of the encoding protein. As a result, synthesis of α-2,6 SA galactose was inhibited. Adsorption of influenza virus particles to the surface of cells transfected with appropriate specific siRNAs was significantly reduced. Intracellular viral genome copy number and virus titer within the supernatant of cells transfected with siRNAs was significantly reduced in a dose-dependent manner compared with those for untransfected cells and cells transfected with non-specific siRNAs.

**Conclusions:**

We used siRNAs targeting ST6GAL1 to inhibit the expression of certain cell surface receptors, thereby preventing virus adsorption. This resulted in the inhibition of human influenza virus infection. Our findings are a significant development in the identification of potential new anti-influenza drug targets.

## Background

Influenza virus infections are considered a significant public health problem given that they cause seasonal epidemics and recurring pandemics [1]. There were more than 29,000 cases of infections, and 3,000 human deaths attributed to the 2009 global H1N1 influenza pandemic [2]. Because of the high mutation rates of the viral genome, vaccines and drugs initially directed against the virus often become ineffective [3,4]. Therefore, measures are urgently needed to prevent and treat influenza virus infections, especially for high-risk groups and in the event of another pandemic. Certain host cell factors involved in the viral infection cycle have attracted interest as therapeutic targets because these are crucial for viral infections. Targeting these factors might inhibit infection, and there is the added advantage that they are less prone to mutations [5-7].

Sialic acid (SA) molecules, found at the non-reducing terminal position of glycoproteins or glycolipids on the surface of cells, are binding targets for influenza A virus (IAV) hemagglutinin (HAs) [8]. The HAs of human IAVs preferentially bind to α-2,6 linked SA, which is abundantly expressed in the human respiratory tract. The HA proteins of avian IAVs prefer α-2,3 linked SA as a receptor, as it is predominant in the avian enteric tract [9]. The binding of HA to its appropriate receptor is crucial for the initiation of infection and therefore serves as a potential therapeutic target. The novel sialidase fusion protein, DAS181 (Fludase), enzymatically removes SAs from the respiratory epithelium and exhibits potent antiviral activity against influenza A and B viruses [10]. Sialyltransferases are key enzymes that regulate the biosynthesis of sialylated oligosaccharide sequences [11]. Weinstein *et al.* concluded that one enzyme, βgalactoside α2,6sialyltransferase I (ST6Gal I), encoded by ST6GAL1, was responsible for the addition of α-2,6 SAs to the Galβ1-4GlcNAc disaccharide found on the glycans of N-linked and some O-linked glycoproteins [12]. Lin *et al.* found that antisense-oligodeoxynucleotides targeting ST6GAL1 mRNAs could inhibit the enzymatic activity of ST6Gal I, and reduced 2,6-sialylation at the cell surface [13,14].

Numerous studies involving small interfering RNAs (siRNAs) in the treatment of viral infections have been conducted [15-17], including our successful application of siRNAs to treat severe acute respiratory syndrome (SARS)-infected rhesus macaques [18]. Qe *et al.* used siRNAs specific for conserved regions of the influenza virus genome; these proved to be potent inhibitors of influenza virus replication *in vitro* and *in vivo*[19,20]. However, siRNAs solely targeting the genes of influenza viruses are unlikely to be sufficient in eliminating infection because there is a high possibility of generating resistant mutants. Therefore siRNAs targeting host cellular determinants crucial for viral entry and/or replication could be a more efficacious antiviral therapy.

Our study was designed to evaluate siRNAs targeting ST6GAL1 in airway epithelial cells. We designed four siRNA duplexes and screened their ability to down-regulate ST6GAL1 expression and affect the biosynthesis of α-2,6-linked SA in human respiratory tract epithelium. The antiviral effects of selected siRNA duplexes were demonstrated using viral binding assays, and by determining cytoplasmic viral genome copy numbers and virus titers in culture supernatants.

## Results

### ST6Gal Ι expression in siRNA-transfected A549, HBE, and HEp-2 respiratory epithelial cells

**Figure 1:**
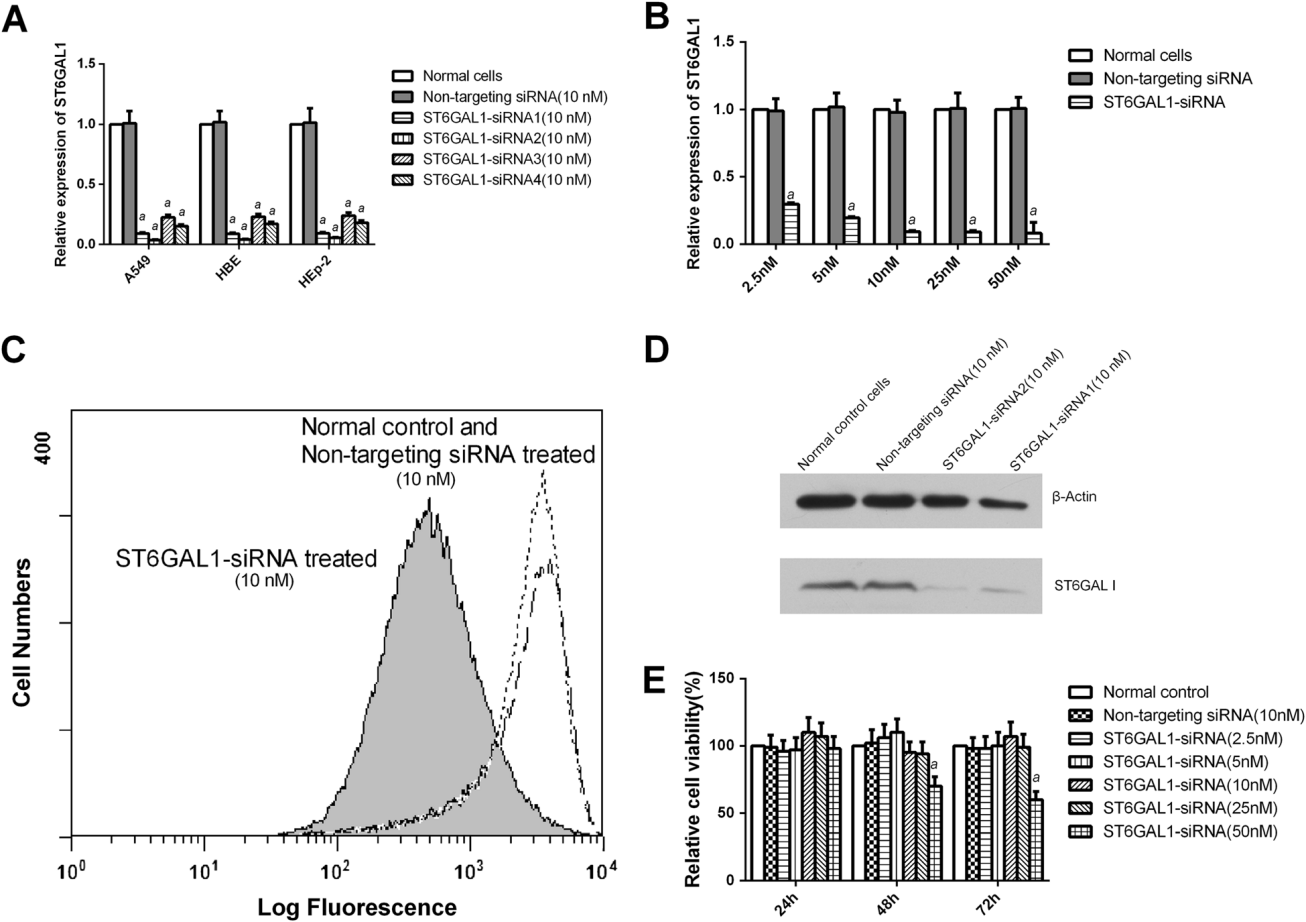
**ST6GAL1-specific siRNAs inhibited ST6Gal 1 expression and SAα2,6Gal synthesis.** Respiratory epithelial cells were transfected with control or ST6GAL1 siRNAs **(A)** At 48 h post-transfection, ST6GAL1 mRNA expression levels were quantified. **(B)** Candidate siRNAs reduce ST6GAL1 expression in a dose-dependent manner. **(C)** FACS analysis showed a decrease in SAα2,6Gal expression on ST6GAL1 siRNA-treated cell surfaces. **(D)** Inhibition of ST6Gal Ι expression due to ST6GAL1 siRNAs. **(E)** We used ST6GAL1 siRNAs at various concentrations and they were not cytotoxic, except at 50 nM. ^a^*P* < 0.05.

Using qPCR assays, we determined there was an 80–90% reduction in ST6GAL1 mRNA expression levels up to 48 h post-transfection (Figure 1A,B). Both ST6GAL1-siRNA01 and −02 were more potent than the other siRNA candidates. Their effects were verified using western blot assays. In cells transfected with the selected ST6GAL1 siRNAs, ST6Gal Ι expression was substantially lower than in those transfected with control siRNAs and untransfected cells (Figure 1D). This roughly corresponds to the reduction in mRNA levels as observed by qPCR.

### Transduced epithelial cells showed normal levels of viability

The concentration of ST6GAL1 siRNAs we used (2.5–25 nM) did not adversely affect the number of viable cells, nor affect cell morphology (data not shown) of A549, HBE, and HEp-2 cells (Figure 1E and Additional file 1: Figure S1). When we used siRNAs at 50 nM some cytotoxicity was observed. Based on these results, we used siRNAs at 10 nM in the remainder of our experiments.

### Down-regulation of influenza virus receptors SAα2,6Gal

**Figure 2:**
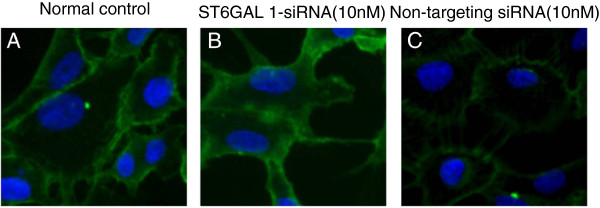
**Treatment with ST6GAL1 siRNAs inhibited SAα2,6Gal expression on cell surfaces.** A549 cells were treated with ST6GAL1 or control siRNAs. SAα2,6Gal was stained with SNA-FITC (green) and cell nuclei were counterstained with DAPI (blue). **(A)** Untreated cells. **(B)** Control siRNAs. **(C)** ST6GAL1 siRNAs.

Expression of SAα2,6Gal receptors was observed on the surface of cultured human A549 cells (Figure 2A). Treatment with ST6GAL1 siRNAs significantly reduced the number of SAα2,6Gal receptors at the cell surface (Figure 2C) compared with control siRNAs (Figure 2B) and untransfected cells (Figure 2A). Similar results were seen for HBE and HEp-2 cells (Additional file 1: Figure S2). Our FACS analysis of A549 cells revealed that ST6GAL1 siRNA-transduced cells had an 89% decrease in SAα2,6Gal expression. Cells treated with control siRNAs and untreated cells displayed normal levels of SAα2,6Gal expression (Figure 1C).

### Receptor specificity of viruses

**Figure 3:**
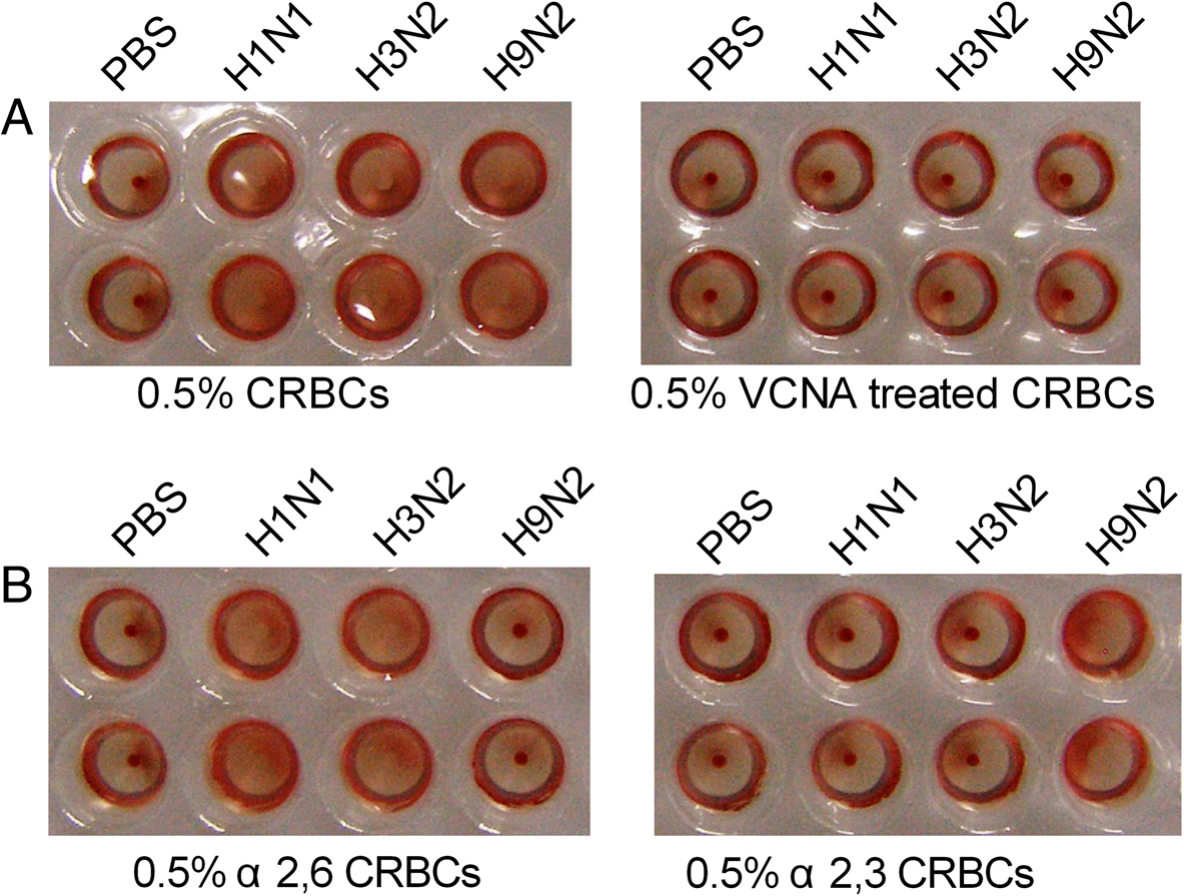
**Receptor specificity of virus strains. (A)** Unmodified (left) and VCNA-treated CRBCs (right). **(B)** SAα2,6Gal-resialylated CRBCs hemagglutinate the H3N2 and pdmH1N1 viruses (left). SAα2,3Gal-resialylated CRBCs hemagglutinate the H9N2 virus (right). Top: two hemagglutination units. Bottom: 1:2 dilution.

All virus strains tested bound to CRBCs, which expressed both α2,3- and α2,6-linked SA. The H9N2 virus preferentially bound to SAα2,3Gal-resialylated CRBCs, whereas the human H1N1 and seasonal human H3N2 influenza virus preferentially bound to the SAα2,6Gal-resialylated CRBCs (Figure 3).

### siRNA-transduced respiratory cells were resistant to viral challenge

**Figure 4:**
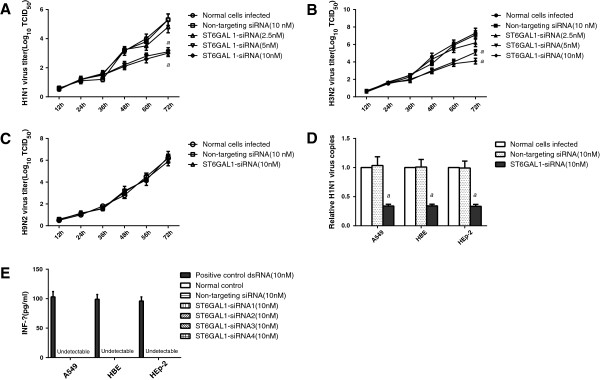
**ST6GAL1 siRNA-transduced respiratory cells resisted human influenza virus challenge and did not induce an interferon response.** Transduced A549 cells were challenged with H3N2, pdmH1N1, or H9N2 viruses. **(A)** A reduction in viral yield was seen in ST6GAL1 siRNA-transduced cells infected with and pdmH1N1 **(B)** H3N2 influenza viruses. ^a^*P* < 0.05. **(C)** Viral yield was not affected when cells were infected with the avian H9N2 virus. **(D)** Treatment with ST6GAL1 siRNAs resulted in a reduced capacity for viral replication during virus entry. ^a^*P* < 0.05. **(E)** ELISAs were used to measure levels of IFN-β production following treatment with siRNAs.

A reduction in viral yield was seen in ST6GAL1 siRNA-transduced A549 cells challenged with the H3N2 and pdmH1N1 strains as compared with control cells (Figure 4A,B). Similar results were observed for HBE and HEp-2 cells (Additional file 1: Figure S3). No differences were observed when cells were infected with the avian H9N2 virus (Figure 4C).

### Inhibition of ST6GAL1 expression affects virus binding and internalization

**Figure 5:**
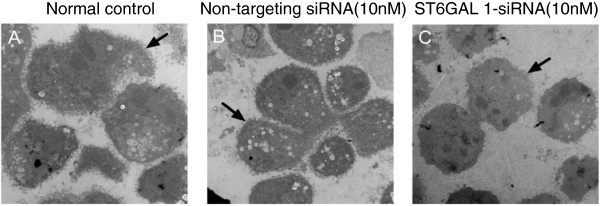
**Virus particle binding assays.** Virus particles binding to the surface of untransfected cells (**A**, black arrow), and cells treated with control siRNAs (**B**, black arrow). The binding of virus particles to the cell surface was adversely affected by treating with ST6GAL1 siRNAs (**C**, black arrow).

Virus particles were abundant on the surface of A549 cells transfected with control siRNAs, and those infected with viruses (Figure 5A,B). However, there was a reduction in the number of bound virus particles for cells treated with ST6GAL1 siRNAs (Figure 5C). The genome copy number of viruses was reduced following transfection of the various cell lines (A549, HBE, and HEp-2) with ST6GAL1 siRNAs (Figure 4D) prior to viral infection.

### The tested siRNAs did not induce an interferon response

The expression of IFN-β in supernatants of siRNA-transfected cell lines (A549, HBE and HEp-2) was not detected. As a positive control, a long double-stranded RNA that is known to induce the expression of IFN-β was included (Figure 4E).

## Discussion

In our study, we were able to demonstrate that down-regulation of the major influenza receptor, SAα2,6Gal, in respiratory epithelial cells was a promising approach to prevent viral entry and establishment of an infection. Most human influenza infections are caused by virus strains transmitted *via* an airborne route from the human respiratory tract, although there are some reports of avian influenza viruses directly infecting humans [21,22]. We designed siRNAs targeting ST6GAL1, in an attempt to inhibit pdmH1N1 and H3N2 virus infection in HEp-2, HBE, and A549 cells, which are representative of the upper, middle and lower respiratory tract epithelial cells, respectively, without inducing an interferon response.

Treatment with siRNAs is not dependent upon a functional immune system. Therefore siRNA therapies could be as effective in elderly or immunocompromised individuals as in immunocompetent individuals [23]. The siRNAs targeting ST6GAL1 that we used in this current study could be ideal in preventing influenza infection in patient groups with low immunity. Our results pertaining to virus binding indicate that ST6GAL1-specific siRNAs reduce the number of IAV virions that attach to epithelial cells, because of reduced expression of SAα 2,6Gal on the cell surface.

Recent studies have suggested that some siRNAs could have side effects [24] that adversely affect cell viability. We demonstrated that the effective dose (10 nM) of siRNAs, under the conditions tested, was not toxic to respiratory epithelial cells *in vitro*. However, we did notice that expression levels of receptors were substantially diminished as a result of siRNA targeting. Influenza viruses naturally infect epithelial cells in the upper respiratory tract and the lungs of humans. Thus, siRNAs can be administered by inhalation. This would result in much higher local siRNA concentrations than could be achieved by parenteral injection, without adversely affecting epithelial cells [23]. Studies focusing on these aspects are currently underway in our laboratories.

In other studies, investigators found that human influenza viruses can still infect ST6GAL1 knock-out mice, achieving similar titers in the lung and trachea as compared with wild-type animals [25]. A possible explanation for this is that there was greater efficiency of infection as a result of a deficient systemic influenza-specific humoral response in these ST6GAL1 knock-out mice [26]. There are two major types of SAα2,3Gal, which differ in their penultimate bond (Neu5Acα2-3Galβ1-3GalNAc or Neu5Acα2-3Galβ1-4GlcNAc) and these are synthesized by different enzymes [27-29]. Some human influenza virus strains propagated in allantoic cavities are able to bind to both SAα2,6Gal and SAα2,3Gal [9,25,30]. When recombinant rat α2,3-sialyltransferase was used to reconstitute sialic acids, only one type of galactose was linked to other glycans through β-1,3 but not β-1,4 linkages [31-33]; however, it is possible that other strains maintain the ability to bind to Neu5Acα2-3Galβ1-4GlcNAc. Thus, SAα2,3Gal (Neu5Acα2-3Galβ1-4GlcNAc) present in these mice can compensate for the loss of SAα2,6Gal [34]. Monteerarat *et al.*[35] reported that suppression of SAα2,3Gal expression by siRNAs against another sialyltransferase, β-galactoside α-2,3-sialyltransferase 4 (ST3GAL4) could inhibit H5N1 avian influenza virus infection. The findings from our current study support the conclusions of Monteerarat *et al*., and we have further extended these to airway epithelial targets of human IAV infection. A combination of these two types of siRNAs might result in broader spectrum synergistic activities, depending upon their targets.

## Conclusions

We demonstrated the *in vitro* efficacy of siRNAs targeting ST6GAL1 in respiratory epithelial cells for the prevention of IAV infections at the virus entry stage. Further *in vivo* preclinical testing is required to determine the suitability of these siRNAs for use in humans.

## Methods

### Cells and viruses

We used Madin–Darby canine kidney (MDCK) cells for virus propagation and 50% tissue culture-infective dose (TCID_50_) titration assays. MDCK cells were in minimal essential medium (MEM; Gibco, Carlsbad, CA, USA) supplemented with 10% fetal bovine serum (FBS; Gibco), 100 U/mL penicillin, and 100 μg/mL streptomycin (Gibco) at 37°C/5% CO_2_. The A549 human lung carcinoma, human bronchial epithelium (HBE), and human laryngeal epidermoid carcinome (HEp-2) cells were used for transfection experiments. These cells were maintained in Dulbecco’s modified Eagle’s medium (DMEM; Gibco) supplemented with 10% FBS, 100 U/mL penicillin, and 100 μg/mL streptomycin at 37°C 5% CO_2_. We used a 2009 pandemic influenza A (H1N1) virus (pdmH1N1), strain A/Guangzhou/GIRD07/2009 (GenBank Accession No. HM_014326-HM_014333). A seasonal H3N2 influenza A virus (A/Guangdong/520/2009) was isolated from a patient with influenza-like symptoms. An influenza A H9N2 isolate (A/Chicken/Guangdong/SS/94) was kindly provided by South China Agricultural University. The pdmH1N1 and H3N2 viruses were grown in MDCK cells at 35°C, while the H9N2 virus was propagated in allantoic cavities of 10-day-old embryonated hens’ eggs at 37°C. All experiments with pdmH1N1 and H9N2 viruses were conducted under biosecurity level three conditions, and higher.

### Preparation and transfection of siRNAs

The siRNAs against ST6GAL1 were designed using BLOCK-iT™ RNAi Designer [36] and synthesized by Invitrogen ( Invitrogen, Carlsbad, CA, USA). Sequences are available in Additional file 1: Table S1. As a negative control, we used non-targeting Allstars® siRNAs (Qiagen, Valencia, CA, USA). All siRNA duplexes were double-stranded RNA molecules comprising 21 nt with a dTdT overhang at the 3’ ends [37]. Target sequences were subjected to a Basic Local Alignment Search Tool (BLAST) search against GenBank to ensure they were unique to ST6GAL1. Airway epithelial cell lines (A549, HBE, and HEp-2) were transfected with either ST6GAL1 or non-targeting Allstars® siRNAs using Lipofectamine® RNAiMax (Invitrogen) according to the manufacturer’s instructions. At different time points post-transfection, cells were either infected with influenza virus or harvested for downstream experiments.

### Detection of ST6GAL1 and ST6Gal I

Expression of ST6GAL1 was detected using real-time reverse transcription polymerase chain reaction (qPCR) assays. Expression of the ST6Gal I protein was determined by western blotting. Respiratory epithelial cells (A549, HBE, and HEp-2) were transfected with control or ST6GAL1 siRNAs (2.5–50 nmol). At 48 h post-transfection we used an RNeasy Mini kit (Qiagen) for RNA extraction according to the manufacturer’s instructions. The extracted total RNA (500 ng/sample) was then used for cDNA synthesis. The resulting cDNA was amplified in a 20-μL reaction containing ST6GAL1-specific forward (0.25 μmol) and reverse (0.25 μmol) primers (Additional file 1: Table S2), and 1× Power SYBR Green PCR Master Mix (Applied Biosystems, Foster City, CA, USA). Reactions were subjected to thermal cycling with an IQ5 System (Bio-Rad, Hercules, CA, USA) involving an initial 10-min denaturation step at 95°C, followed by 40 cycles of 95°C for 15 s and 60°C for 60 s. Fluorescence signals from these reactions were captured at the end of the 60°C extension step for each cycle. To determine the specificity of the assay, amplicons were subject to melting curve analysis after the 40th cycle (65–95°C, 0.1°C/s). Our data were analyzed using the 2^-ΔΔCT^ method, according to the manufacturer’s instructions, with ST6GAL1 expression levels normalized to β-actin mRNA levels.

After transfection for 48 h, A549 cells were lysed in 50 mM Tris–HCl buffer (pH 7.4) containing 1% Triton X-100, 0.5 mM phenylmethylsulfonyl fluoride (PMSF), 20 μg/mL leupeptin, 4 mM sodium fluoride, and 200 μM sodium pervanadate. Protein concentrations in the lysates were determined with a BCA assay kit (Pierce, Rockford, IL, USA). Proteins in lysates were resolved under reducing conditions for sodium dodecyl sulfate polyacrylamide gel electrophoresis (SDS-PAGE). We probed polyvinylidene fluoride (PVDF) membranes with 5 μg/mL of a rabbit antihuman ST6Gal Ι polyclonal antibody (Abcam, Cambridge, MA, USA) followed by a horseradish peroxidase (HRP)conjugated antirabbit IgG secondary antibody(Abcam). Specific signals were visualized using an ECL kit (Pierce). Protein concentrations between wells were normalized using HRP-conjugated β-actin-specific monoclonal antibodies (Sigma-Aldrich, St. Louis, MO, USA).

### Cell viability

Cultured cells in the logarithmic growth phase were trypsinized, seeded into 96-well plates, and transfected with ST6GAL1 (2.5–50 nmol) or control (10 nmol) siRNAs. At 24, 48, and 72 h post-transfection, cell viability was determined using 3-(4,5-dimethylthiazol-2-yl)-2,5-diphenyl-2H-tetrazolium bromide (MTT) assays (Sigma-Aldrich). The absorbance at 492 nm was measured in a spectrophotometer (Molecular Devices, Palo Alto, CA, USA). Background values were subtracted from the average absorbance value obtained for each siRNA treatment and then compared with the value obtained when siRNAs were absent (100% viability). Each assay was performed in duplicate in at least four wells.

### Detection of α-2,6 linked SA on the cell surface

The A549, HBE, and HEp-2 cells were treated with ST6GAL1 or control siRNAs and cultured for 72 h. Cells were washed with phosphate-buffered saline (PBS) and fixed in 3.7% formaldehyde in PBS for 30 min. For detection of sialic acid residues on the surface of cells, apical monolayers were blocked with 3% bovine serum albumin (BSA; Merck, Darmstadt, Germany) in PBS for 30 min and then incubated with 5 μg/mL fluorescein isothiocyanate (FITC)-conjugated *Sambucus nigra* lectin (SNA; Vector Laboratories, Burlingame, CA, USA) for 1 h. To confirm the specificity of lectin binding, monolayers were treated with 50 mU *Vibrio cholerae* neuraminidase (VCNA; Roche, Almere, Netherlands) for 1 h prior to fixation and then examined with a rapid-scanning confocal laser microscope (Nikon Corp, Tokyo, Japan).

### Flow cytometry

Approximately 10^6^ cells transfected with control or ST6GAL1 siRNAs were scraped from the culture surface and washed twice with PBS containing 10 mM glycine, and then washed once with buffer 1 (50 mM Tris–HCl, 0.15 M NaCl, 1 mM MgCl_2_, 1 mM MnCl_2_, 1 mM CaCl_2_, pH 7.5). Cells were blocked with 3% BSA-PBS for 1 h on ice and washed in the same manner as described above. After centrifugation, the cell pellet was incubated with FITC-conjugated SNA at room temperature for 30 min, then washed and fixed with 1% paraformaldehyde. After another three washes with PBS, mean fluorescence intensities were determined on a fluorescence-activated cell sorter (FACS) Calibur flow cytometer (BD, San Jose, CA, USA) by counting a minimum of 10,000 events.

### Receptor specificity of virus strains

To study the receptor-binding properties of the virus strains used, we enzymatically modified chicken red blood cells (CRBCs) to express either sialic acid (SA)-α2,6-Galactose (Gal) or SAα2,3Gal as previously described [38,39] with minor modifications. Briefly, SA was removed from 100 μL of 10% CRBCs using 50 mU VCNA at 37°C for 1 h. Subsequent resialylation was performed using 50 μL of 0.5 mU α2,3-(N)-sialyltransferase (Calbiochem, La Jolla, CA, USA) or 125 μL of 2 mU α2,6-( N)-sialyltransferase (Japan Tobacco, Shizuoka, Japan), and 1.5 mM cytidine monophospho-N-acetylneuraminic (CMP) sialic acid (Sigma-Aldrich) at 37°C for 30 or 60 min, respectively. Receptor specificity of the virus strains was then determined using standard hemagglutination assays with the modified CRBCs.

### Influenza virus challenge of ST6GAL1-siRNA transduced epithelial cells

All challenge experiments were carried out at a multiplicity of infection (MOI) of 0.01 for 1 h in the presence of N-*p*-Tosyl-L-phenylalanine chloromethyl ketone (TPCK)-trypsin (Sigma-Aldrich). Viral supernatants were harvested at various time points post-infection for TCID_50_ assays. To obtain dose–response curves, a dilution series of siRNAs were added to cells in 96-well plates in triplicate. Cells were challenged and supernatants were examined as described above [40].

The titer of viruses in supernatants were determined using TCID_50_ assays as previously described [20]. Briefly, MDCK cells were seeded onto flat-bottom 96-well plates (3 × 10^4^ cells/well); 24 h later, serum-containing medium was removed and 25 μL of virus-containing supernatants (serially diluted ten-fold from 10° to10 ^−8^) was added to wells in triplicate. After incubation for 1 h, 175 μL of infection medium containing TPCK-trypsin (1.25 μg/mL) was added to each well. After incubation for 48 h at 37°C, the presence or absence of virus in culture supernatants was determined by hemagglutination of CRBCs. Virus titers were determined by interpolation of the dilution endpoint that infected 50% of wells. Virus titers are presented as log_10_ TCID_50_.

### Electron microscopy

Cells were transfected with control or ST6GAL1 siRNAs, then infected with virus at an MOI of 50, and chilled at 4°C for 90 min. Infected cells were harvested and washed three times with PBS, then fixed with 3% glutaraldehyde for 45 min at room temperature, and post-fixed with 1% osmium tetroxide. Fixed cells were dehydrated with increasing concentrations of acetone from 30% to 100% and embedded in an epoxy resin. Polymerization was conducted at 60°C for 48 h. Ultrathin sections were stained with uranyl acetate and lead citrate, and sections viewed and photographed with a Hitachi H-800 transmission electron microscope (Hitachi Co., Tokyo, Japan).

### Quantitation of viral genome copies by qPCR

We extracted RNA 2 h after virus infection using a QIAamp RNA isolation kit (Qiagen). First-strand cDNA was synthesized using RNAse H^+^ reverse transcriptase (Invitrogen) and random primers. We then used 2 μL of cDNA for each qPCR assay, along with primers (Additional file 1: Table S2), fluorescent probe, and Master Mix (Applied Biosystems). Samples were subjected to thermal cycling on an IQ5 System (Bio-Rad, Hercules, CA, USA): 42°C for 5 min; 95°C for 10 s; and 40 cycles of 95°C for 5 s and 60°C for 30 s. Expression levels of viral RNAs were normalized to the constitutive expression of ribonucleoprotein. All measurements were conducted three times for statistical analysis.

### IFN-β assays

The A549, HBE, and HEp-2 cells were transfected with either control or ST6GAL1 siRNAs (10 nM). We measured the levels of IFN-β in culture supernatants 24 h later using an enzyme-linked immunosorbent assay (ELISA; PBL Biomedical Laboratories, Piscataway, NJ, USA). A long double-stranded RNA that induced the expression of IFN-β used as a positive control.

### Statistical analysis

All statistical analyses were performed using SPSS 12.0 (SPSS Inc., Chicago, IL, USA). The significance of variability among experimental groups was determined using one-way ANOVA, the paired *t*-test, or the Mann–Whitney U test. All differences were considered statistically significant if the *P*-value was less than 0.05.

## Competing interests

The authors declare that they have no competing interests.

## Authors’ contributions

DW contributed to the study design, data collection, most experiments, writing of the initial draft, and revising the manuscript. WB, YW, WG, and RL collected the preliminary data, and helped to perform some experiments. ZY and NZ participated in the study design, interpretation of the data, the study coordination, technical issues, and revision of the manuscript. All authors read and approved the final manuscript.

## Supplementary Material

Additional file 1: Table S1 Nomenclature of 4 candidate siRNA duplexes targeting ST6GAL1 gene. **Table S2.** Real time RT-PCR primers and probes. **Figure S1.** Cells viability. **Figure S2.** Down regulation of influenza virus receptors SA α 2,6Gal on transduced respiratory epithelium(HBE, Hep-2). **Figure S3.** Targeted siRNA transduced respiratory epitheliums resist influenza virus challenge. Click here for file
