# Supplementary material for: Gene silencing of β-galactosamide α-2,6-sialyltransferase 1 inhibits human influenza virus infection of airway epithelial cells
Source: BMC Microbiol. 2014 Mar 27;14:78. doi: 10.1186/1471-2180-14-78 (PMC3986885; doi:10.1186/1471-2180-14-78)
Supplement: Additional file 1: Table S1 — Nomenclature of 4 candidate siRNA duplexes targeting ST6GAL1 gene. Table S2. Real time RT-PCR primers and probes. Figure S1. Cells viability. Figure S2. Down regulation of influenza virus receptors SA α 2,6Gal on transduced respiratory epithelium(HBE, Hep-2). Figure S3. Targeted siRNA transduced respiratory epitheliums resist influenza virus challenge. [file 1471-2180-14-78-S1.pdf]

Table S1. Nomenclature of 4 candidate siRNA duplexes targeting ST6GAL1 gene

Name	Coding region	Position (nt)	Sequence (5’ – 3’)
ST6GAL1-siRNA1	ORF	1278-1296	CAGCCAACUUCCAACAAGAdTdT, dTdTGUCGGUUGAAGGUUGUUCU;
ST6GAL1-siRNA 2	ORF	1438-1456	GUACCAGAAUCCGGAUUAUdTdT, dTdTCAUGGUCUUAGGCCUAAUA;
ST6GAL1-siRNA 3	ORF	1599-1617	CUGGGAUGCUUGGUAUCAUdTdT, dTdTGACCCUACGAACCAUAGUA;
ST6GAL1-siRNA 4	ORF	1144-1162	CUGGGAUGCUUGGUAUCAUdTdT, dTdTGACCCUACGAACCAUAGUA

Table S2. Real time RT-PCR primers and probes

Name	Sequence (5’ – 3’)
ST6Gal1 forward primer	GAAACCATGCAAAGCAGTGGAC
ST6Gal1 reverse primer	GCCCATTCCCAAGCAGAATC
β-actin forward primer	TGGCACCCAGCACAATGAA
β-actin reverse primer	CTAAGTCATAGTCCGCCTAGAAGCA
The influenza A viru forward primer	GACCRATCCTGTCACCTCTGAC
The influenza A viru reverse primer	AGGGCATTYTGGACAAAKCGTCTA
The influenza A viru fluoresce probe	TGCAGTCCTCGCTCACTGGGCACG-FAM
RnaseP Forward primer	AGATTTGGACCTGCGAGCG
RnaseP Reverse primer	GAGCGGCTGTCTCCACAAGT
RnaseP fluoresce probe	TTCTGACCTGAAGGCTCTGCGCG-FAM

Figure S1. Cells viability

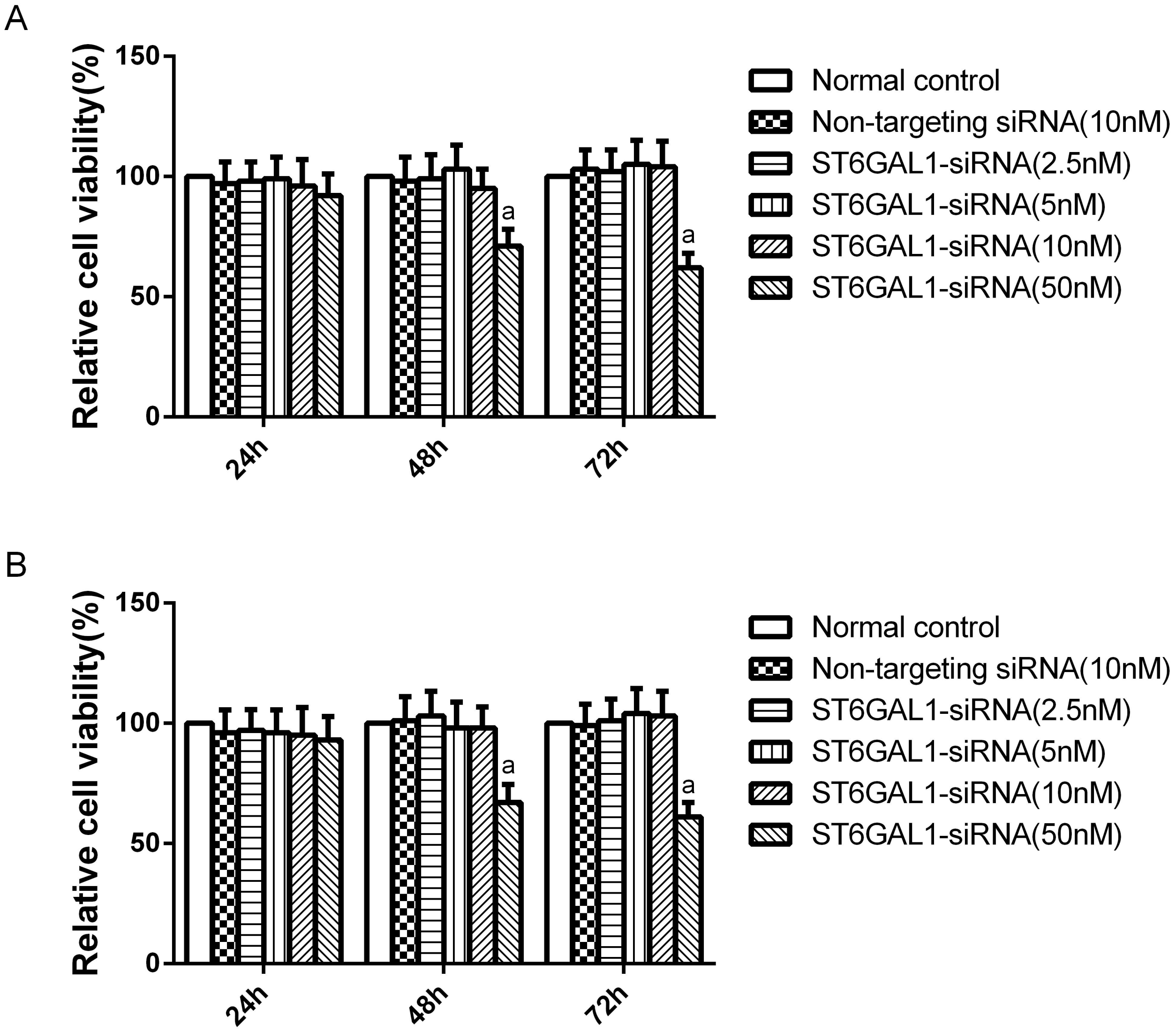

MTT assays for the influence of the siRNAs on the viability of epitheliums. HBE (A) and HEp-2 (B) cells were treated with ST6Gal1-siRNA(2.5-50 nmol) or Non-targeting siRNA . MTT tests were performed at 24, 48, 72h post-transfection. Background values were subtracted from the absorbance average value (four wells) obtained for each siRNA treatment and then compared with that scored in the absence of siRNA (100% viability). Each assay was performed in duplicates of at least four wells. ST6Gal 1-siRNA(2.5-10 nmol) is not toxic to the cells, but small toxicity was observed for the highest siRNA concentration(50nM) $^{a}P<0.05$

Figure S2. Down regulation of influenza virus receptors SA α 2,6Gal on transduced respiratory epithelium（HBE, Hep-2）

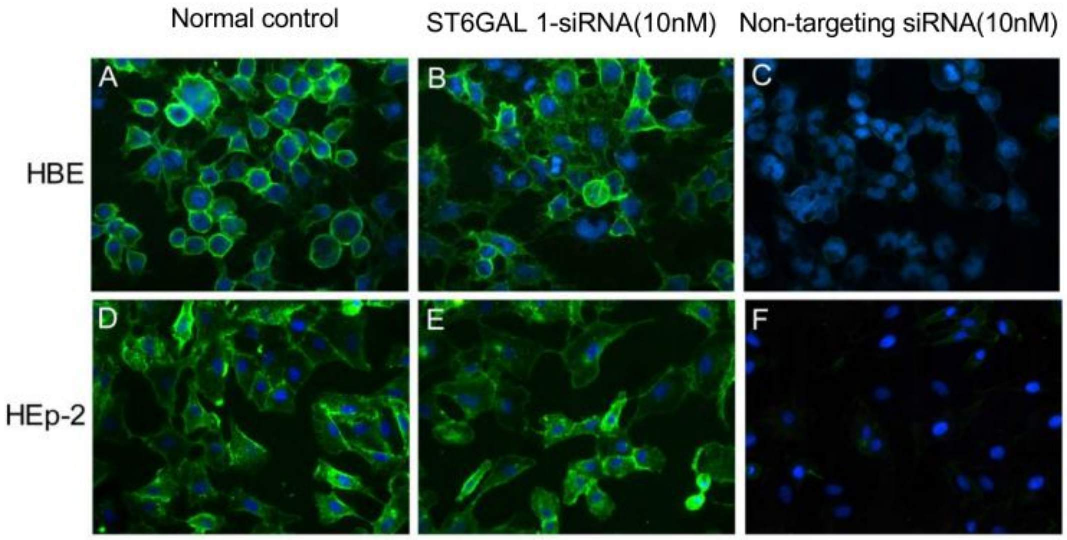

Fluorescence microscope analysis of the expression of SAα2,6Gal on the surface of epithelium. HBE (top panel) and HEp-2 (below panel) cells were treated with either ST6GAL1-siRNA or Non-targeting siRNA at final concentrations of 10nM. SA α2, 6Gal was stained with SNA-FITC (green fluorescence), cell nuclei were counterstained with DAPI, and then cells were subjected to fluorescence microscope analyses. ST6GAL1-siRNA treated cells(C, F) demonstrated lower level of fluorescence intensity compared to Non-targeting siRNA treated (B, E) and normal control cells (A, D).

Figure S3. Targeted siRNA transduced respiratory epitheliums resist influenza virus challenge

A

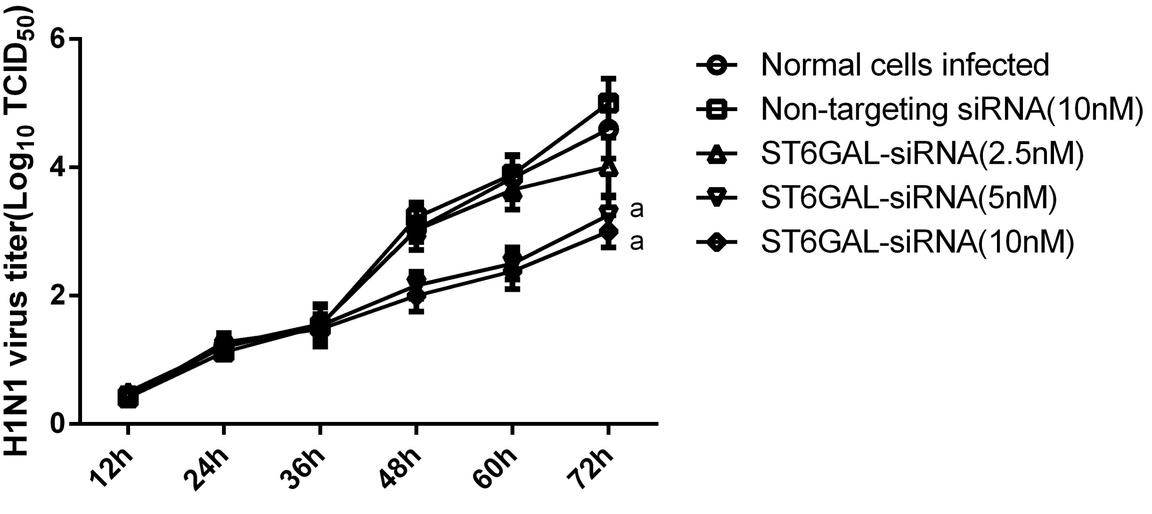

B

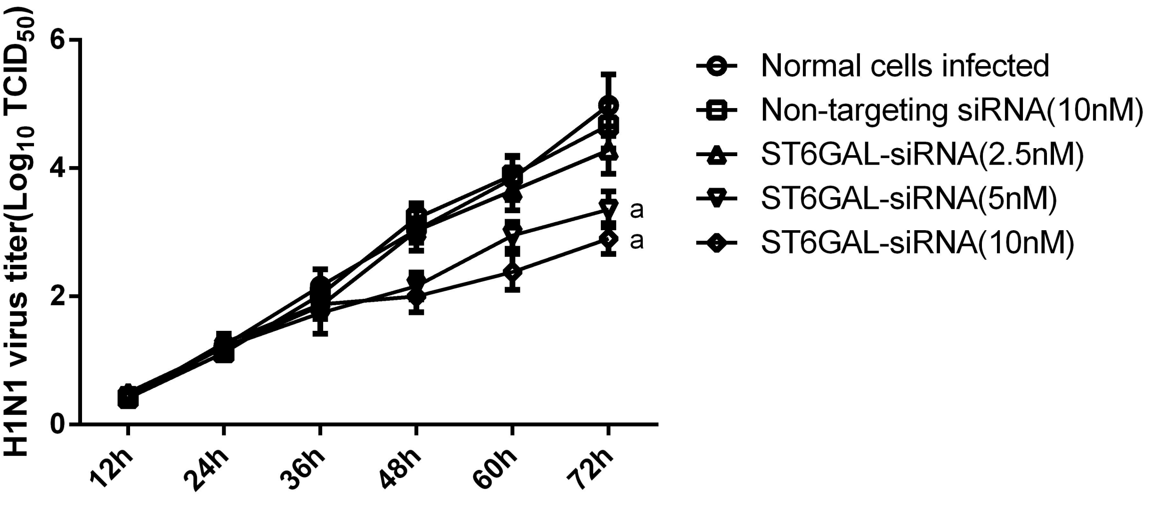

Influenza virus resistance of ST6Gal1-siRNA transduced epitheliums. Transduced HBE (A) and HEp-2 (B) cells were challenged with pdmH1N1 virus. $TCID_{50}$ assays were performed on culture supernatants taken at various time points post-infection. Experiments were performed in triplicate. A significant decrease in viral titer was observed in cells transfected with ST6Gal1-siRNA as compared to cells transfected with Non-targeting siRNA or normal cells and in a dose-dependent manner ($^{a}P<0.05$, Student's t test).
